# A Young Woman with Paraneoplastic Cushing’s Syndrome Due to a Pulmonary Carcinoid

**DOI:** 10.3390/reports8040226

**Published:** 2025-11-03

**Authors:** Marine Sluys, Pauline Delannoy, Laurence Lousberg, Marie Strivay, Adrian F. Daly, Patrick Pétrossians

**Affiliations:** 1 Departments of Diabetology, CHU de Liège, 4000 Liège, Belgium; 2 Department of Endocrinology, CHU de Liège, 4000 Liège, Belgium; 3 Department of Oncology, CHU de Liège, 4000 Liège, Belgium; 4 Department of Endocrinology and Diabetology, CHR Citadelle, 4000 Liège, Belgium

**Keywords:** Cushing’s syndrome, ectopic ACTH, neuroendocrine tumor, pulmonary carcinoid

## Abstract

**Background and Clinical Significance:** Ectopic ACTH secretion is a rare, potentially life-threatening cause of Cushing’s syndrome that can be overlooked when small neuroendocrine tumors evade standard imaging. **Case Presentation:** A 34-year-old woman presented with rapidly progressing clinical signs/symptoms of Cushing’s syndrome and demonstrated marked hypercortisolism (cortisol 2428 nmol/L; ACTH 163 ng/mL; urinary free cortisol 815 μg/24 h; K+ 2.4 mmol/L). Small hypermetabolic nodules were noted in her right lung on ^18^F-FDG PET/CT but were initially deemed to be infectious; DOTANOC PET-CT and inferior petrosal sinus sampling were non-diagnostic. After medically induced inhibition of cortisol, repeat PET/CT showed a persistent 13 mm lung nodule. Biopsy confirmed a well-differentiated pulmonary carcinoid (Ki-67 3%), and lobectomy achieved biochemical remission. **Conclusions:** Diagnostic delay stemmed from human factors despite early suggestive imaging. Ectopic ACTH secretion should remain high on the differential diagnosis in rapidly evolving, severe ACTH-dependent Cushing’s disease; early, decisive diagnosis and coordinated care overseen by endocrinologists—preferably in expert centers—can shorten exposure to deleteriously high cortisol levels and improve outcomes.

## 1. Introduction and Clinical Significance

Ectopic ACTH secretion accounts for 10–15% of ACTH-dependent Cushing’s syndrome (CS) but progresses rapidly and carries excess cardiovascular (including thromboembolic) and infectious mortality. Tumors are frequently small and might evade first-line imaging, leading to diagnostic delays. Non-awareness of the condition among general medical staff might also delay the diagnosis. Our report illustrates that this pathology needs to be familiar to physicians and that endocrinologists play a central role in directing the diagnosis, particularly in expert centers.

Endogenous CS is a rare condition with an annual incidence of 2–3 cases per million [1]. It results from prolonged exposure to excess corticosteroids and presents with a characteristic clinical phenotype, including facial changes (moon facies), a buffalo hump fat pad, and purple striae. Metabolic and catabolic consequences include hypertension, hypokalemia, severe hyperglycemia, and muscle wasting [2]. Patients also face increased risks of infection, sepsis, venous thrombosis, cardiac and respiratory failure, gastric perforation, pancreatitis, and psychiatric changes [2].

CS can be ACTH-dependent (70–80% of cases) or ACTH-independent (20–30%) [3]. Among ACTH-dependent cases, 80–90% are caused by pituitary adenomas (Cushing’s disease), while 10–20% result from ectopic ACTH secretion, often due to bronchial, thymic, or pancreatic neuroendocrine tumors (NET), pheochromocytomas, or small-cell carcinomas [3]. Identifying the ACTH source is challenging because these NETs are frequently small and slow growing [4]. Pulmonary carcinoids are the most common site for ectopic ACTH-secreting lesions, and more than half of these are small, measuring < 2 cm in diameter. [5]. Delayed localization of ectopic ACTH-secreting lesions prolongs exposure to deleterious cortisol levels, worsening morbidity. Many of these tumors are small and difficult to localize. In contrast, endogenous ACTH-independent CS typically arises from autonomous cortisol secretion by adrenal tumors—usually benign adrenocortical adenomas (2/3 of cases), adrenocortical carcinomas (1/3), or rare bilateral adrenal disorders like pigmented micronodular adrenal hyperplasia, seen in diseases like Carney complex [6].

The management of CS requires a structured approach: confirming hypercortisolism, identifying the etiology and source of excess hormone secretion, assessing severity and complications, initiating treatment, and long-term follow-up [7]. Here, we describe a young woman whose tumor, although present on imaging, was not immediately targeted due to an alternate initial radiological diagnosis, which complicated the diagnostic and therapeutic process.

## 2. Case Presentation

A 34-year-old female presented to an outpatient clinic at another center with a six-month history of weight gain, facial plethora, severe proximal myopathy, and alopecia. Past medical history included recent-onset depression, which was treated with olanzapine. Physical examination revealed a moon face, a dorsocervical fat pad, and purple abdominal striae. CS was suspected, and initial laboratory tests showed hypokalemia (2.4 mmol/L) and hypercortisolemia at 2427.58 nmol/L (normal range (NR): 275–685 nmol/L). Due to her debilitated state and biochemical status, she was admitted to that hospital, and her potassium was supplemented intravenously. An elevated ACTH at 163 ng/mL (NR: 5–60 ng/mL) and urinary free cortisol (815 μg/24 h, NR: 36–137 μg/24 h) supported a likely diagnosis of ACTH-dependent CS. No lesion was seen on 1.5 Tesla pituitary magnetic resonance imaging (MRI), and abdominal/pelvic computed tomography (CT) was also unremarkable. ^18^F fluorodeoxyglucose (FDG) positron emission tomography (PET)/CT identified a small (8 mm) hypermetabolic pulmonary nodule in the lower right lobe and two hypermetabolic micronodules (1 cm and 3 mm in diameter) in the upper right lobe. A DOTANOC PET-CT did not identify these hot spots. A differential diagnosis of ectopic ACTH secretion by a pulmonary NET was proposed by the endocrine staff at that hospital. However, at the time, the radiological and pulmonary opinions favored an infectious source over neoplasia, and the possibility of aspiration pneumonia was investigated. Indeed, otorhinolaryngological evaluation showed swallowing dysfunction with hypotonia and oral-lingual-facial incoordination, requiring discontinuation of oral feeding. Further investigations for a small pituitary lesion were undertaken, but a second pituitary MRI (3 Tesla) was normal. A corticotropin-releasing hormone (CRH) stimulation test and inferior petrosal sinus sampling were non-contributory, as ACTH levels were low in all samples during the test. ACTH levels measured later in peripheral blood remained elevated, suggesting sampling problems. A long (48 h) dexamethasone suppression test did not result in any suppression of serum cortisol.

**Figure 1:**
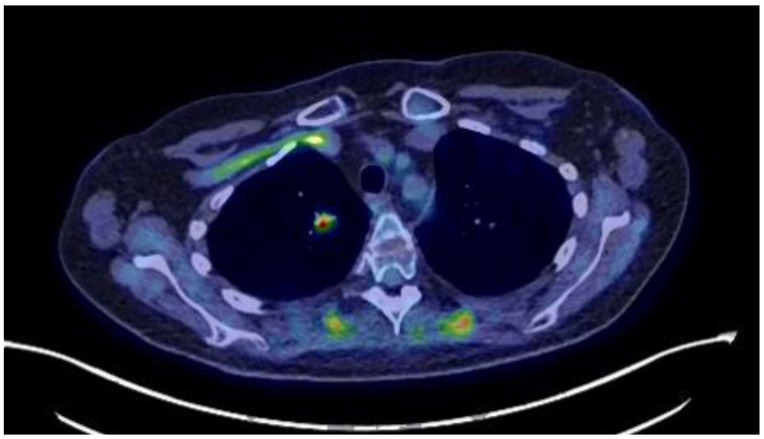
^18^F FDG PET/CT showing a hypermetabolic region in the right lung.

**Figure 2:**
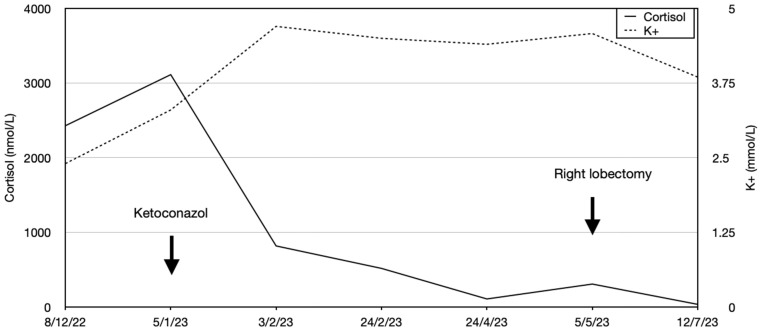
Evolution of cortisol and potassium levels following diagnosis and during treatment.

The patient developed impaired alertness, tetrapyramidal syndrome, and myopathy, and a T8 osteoporotic compression fracture was identified. Mild hypokalemia was still present (3.47 mmol/L). The endocrine team requested a biopsy of the pulmonary lesions to seek an ectopic source of ACTH, but other specialties maintained that the pulmonary lesions were of infectious origin, and a biopsy was not performed. Nevertheless, ketoconazole (escalated from 600 to 800 mg/day) and hydrocortisone (10 mg/day) were started with liver-enzyme monitoring. This reduced cortisol by about 50% from baseline, although hypercortisolemia remained present, and the hypokalemia resolved. The patient’s clinical condition improved, and she was transferred to our institution for physiotherapy and rehabilitation. A referral to our endocrine group led us to reinvestigate the pulmonary lesions, and a new ^18^F-FDG-PET/CT showed the persistence of a 13 mm hypermetabolic nodule (SUVmax: 6.2) in the right lower lobe (Figure 1). On our recommendation, a CT-guided biopsy was performed, which was suggestive of a carcinoid lesion. She was referred for thoracic surgery, and a right lower lobectomy with systematic lymph node dissection was performed. This permitted an R0 resection, and ketoconazole was discontinued (Figure 2). Pathology showed a 1.6 × 1.5 × 1.5 cm, carcinoid-type, typical lung NET (Ki-67: <2%; no necrosis; Thyroid Transcription Factor 1 (TTF-1): intensely positive; CD56: intense, diffuse positivity; keratin-7 partial positivity). As expected, the patient developed ACTH deficiency postoperatively and was treated with hydrocortisone 20 mg/day, with a taper over nine months. At the last follow-up, she was biochemically and hormonally normal, and the signs and symptoms of CS, including depression, had regressed. Long-term treatment involved continued physical rehabilitation, oral calcium/vitamin D, and iron treatment.

## 3. Discussion

Ectopic ACTH secretion is a very rare but critically important cause of endogenous CS that is associated with a female predominance [8]. It is usually associated with NETs, most commonly bronchopulmonary carcinoids (in ~50% of cases). The current case had features consistent with a low-grade typical lung-NET (Ki-67 > 2%, no necrosis) according to the current WHO criteria [9]. Clinical suspicion should be high in patients with rapid-onset hypercortisolism, markedly elevated ACTH, severe hypokalemia, early-onset hypertension, and the presence of psychiatric symptoms, diabetes, and osteoporosis with fractures [2].

Although there is no universally accepted diagnostic algorithm, iatrogenic Cushing’s syndrome must first be ruled out. Hormonal testing is a linchpin of the initial diagnostic workup of individuals with suspected CS. Urinary free cortisol (UFC) is a good marker of biologically active cortisol, but its high variability often requires repeated measurements. UFC may be falsely elevated with polyuria or falsely normal in renal failure. Simultaneous creatinine assessment helps validate collection completeness. Midnight salivary cortisol measurement is a non-invasive technique with high sensitivity, though gingival bleeding may lead to false positives. A cortisol value <50 nmol/L typically excludes hypercortisolism [1]. In the presence of confirmed hypercortisolism, plasma ACTH levels help guide the search for the origin: low levels suggest an adrenal cause, and normal or elevated values point to pituitary or ectopic sources. ACTH concentration below 10 pg/mL on two occasions suggests primary hypercortisolism. Levels higher than 20 pg/mL orient toward ACTH-dependent CS. Values between these extremes, 10 and 20 pg/mL, represent a “grey zone” of overlap between primary adrenal hypercortisolism and ACTH-dependent CS [9]. The 1 mg overnight dexamethasone suppression test is useful for screening; a post-dose morning cortisol >50 nmol/L suggests hypercortisolism. Longer (48h) dexamethasone suppression tests and dynamic tests with CRH or desmopressin can help to distinguish Cushing’s disease from ectopic ACTH syndrome. Ectopic sources typically do not respond to these stimuli, as seen with the dexamethasone suppression test results in this case [10].

MRI of the pituitary is essential in determining the origin of ACTH-dependent CS. Pituitary corticotropinomas are often slightly hypointense in T1 and hyperintense in T2 [11]. Small tumors are, however, not always detected. Even after gadolinium injection, a microadenoma may be masked by enhancement of normal surrounding pituitary tissue [11]. An adenoma is detected in approximately 50 to 70% of cases, given that 50% of corticotropic adenomas are macroadenomas or microadenomas larger than 5 mm [10]. In addition, MRI is an important element in planning the neurosurgical approach. Whole-body imaging (CT/MRI) and functional imaging such as DOTANOC PET-CT should be performed if pituitary MRI is inconclusive, as they can pinpoint bronchial or pancreatic NETs [2]. It should be noted that the spatial resolution of certain PET-CT modalities is 1 cm, so smaller lesions might not be well visualized [12]. FDG PET-CT is valuable in identifying metabolically active neuroendocrine tumors and evaluating treatment response. Repeating imaging every 6 months is recommended when no lesion is initially identified.

Inferior petrosal sinus sampling (IPSS) remains the gold standard to differentiate pituitary from ectopic sources when imaging is equivocal. A central-to-peripheral ACTH gradient >2 supports a pituitary origin [10]. This invasive procedure must be performed in experienced centers due to the risk of complications. In the current case, IPSS was inconclusive due to unmeasurable ACTH levels, suggesting a handling or laboratory error.

Severe Cushing’s syndrome is defined by serum cortisol >1000 nmol/L or UFC levels five times the upper limit of normal [13] and represents a medical emergency. The primary treatment for paraneoplastic CS is complete surgical resection of the ACTH-secreting tumor. When surgery is not feasible—due to occult, inaccessible, or metastatic tumors—medical therapy is used to reduce cortisol and control complications. Common agents include ketoconazole and/or metyrapone, which are orally administered. These reduce cortisol production by inhibiting adrenal steroidogenic enzymes [2]. Ketoconazole mainly inhibits the activity of 17α-hydroxylase but also the 11-hydroxylation steps in cytochrome P450. It therefore blocks the synthesis of cortisol and aldosterone by inhibiting the activity of C17-20 lyase in the adrenal glands and Leydig cells. Metyrapone reduces cortisol and aldosterone synthesis by inhibiting the activity of 11β-hydroxylase and, to a lesser degree, 18-hydroxylase. It induces an increase in androgenic and mineralocorticoid precursors (11-deoxycorticosterone, 11-deoxycortisol), which can lead to hirsutism and hypokalemia. Both drugs have a short half-life (2–4 h for ketoconazole and 1–2 h for metyrapone). The main side effects of metyrapone and ketoconazole are gastrointestinal disorders and elevated transaminases, which are reversible on discontinuation of treatment. As these two drugs act synergistically, they can be used together at lower doses to optimize tolerability. Osilodrostat is a newer, potent, orally administered inhibitor of 11 β-hydroxylase. Osilodrostat inhibits the final stage of cortisol synthesis, as well as aldosterone synthase, which catalyzes the conversion of 11-deoxycorticosterone into aldosterone. It can be used in both intensive care units and in outpatients [14]. Although rapid acting, these enzyme inhibitors may not be sufficiently potent for the acute treatment of severe hypercortisolism, and in cases where oral administration is not feasible. Some medications are not approved for use in certain countries [14].

Etomidate is a potent, imidazole-derived, 11 β-hydroxylase inhibitor that is administered intravenously. Originally used as an anesthetic agent, lower doses reduce cortisol. Etomidate can be administered at higher doses in emergency cases, usually in the intensive care setting, with careful dose titration to produce a rapid reduction in cortisol. At lower doses, etomidate can be used to produce a slower decrease in cortisol in appropriate patients [13].

Somatostatin analogs (e.g., lanreotide) combined with cabergoline may suppress ACTH secretion in some NET [14,15]. Bilateral adrenalectomy, while rare, offers definitive control of hypercortisolism but requires lifelong steroid replacement. It has a mortality rate of 0–8% and is an option when other therapies fail, or the tumor remains unidentified or is metastatic. In surgical cases, preoperative control of hypercortisolemia is crucial to reduce postoperative complications such as infection and thrombosis [2]. The role of other treatments like radiotherapy and radio-interventional therapy (radiofrequency, cryoablation, etc.) is still debatable [2]. Close endocrinological follow-up is mandatory after tumor resection. Patients often require hydrocortisone replacement therapy due to tertiary adrenal insufficiency caused by prolonged ACTH suppression.

Prognosis depends on the histological nature of the tumor [16]. Imaging should be repeated three months after surgery, then every six months for 2–3 years, and annually up to five years [16].

## 4. Conclusions

An ectopic ACTH-secreting tumor is a rare but severe cause of CS. These tumors are often small, variably located, and difficult to detect. Rapid recognition and treatment are critical to avoid life-threatening complications. The investigation and management of ectopic CS is replete with potential pitfalls due to the nature of the disease and the interpretation of diagnostic tests.

Here, we note that lack of consensus among experts about the nature of small lesions that are identified on imaging can be an important pitfall. In this case, initial exploration raised the suspicion of paraneoplastic CS. This diagnosis was essentially confirmed after a normal pituitary MRI and a PET/CT showing a hypermetabolic pulmonary lesion. However, after multidisciplinary discussion, this lesion was deemed to be more likely infectious in nature and was not initially biopsied. This led to a second (3 Tesla) pituitary MRI and a petrous sinus sampling. When the patient was transferred to our service, we confirmed the PET/CT findings and obtained a biopsy that confirmed the diagnosis of a Grade 1 NET, which was subsequently completely resected. Two and a half years later, the patient is biochemically normal and asymptomatic. In cases of ACTH-dependent CS, an ectopic cause needs to be at the forefront of the minds of all investigating medical staff, particularly in cases of rapidly evolving CS and major biochemical disturbances.

## Data Availability

The original contributions presented in this study are included in the article. Further inquiries can be directed to the corresponding author.
